# Case Report: Disappearance of Late Gadolinium Enhancement and Full Functional Recovery in a Young Patient With SARS-CoV-2 Vaccine-Related Myocarditis

**DOI:** 10.3389/fcvm.2022.852931

**Published:** 2022-03-08

**Authors:** Grigorios Korosoglou, Peter Nunninger, Sorin Giusca

**Affiliations:** ^1^Department of Cardiology and Vascular Medicine, GRN Hospital Weinheim, Weinheim, Germany; ^2^Cardiac Imaging Center Weinheim, Hector Foundation, Weinheim, Germany; ^3^Department of Radiology, GRN Hospital Weinheim, Weinheim, Germany

**Keywords:** SARS-CoV-2 vaccine, acute myocarditis, late gadolinium enhancement, oedema, fibrosis, T1/T2 mapping

## Abstract

Acute myocarditis was recently demonstrated in previously healthy young male patients after receipt of mRNA SARS-CoV-2 vaccines. Herein, we report on a 21-year-old man who presented with acute fatigue, myalgia, and chest pain 2 days after his second SARS-CoV-2 vaccination with BNT162b2. Cardiac magnetic resonance (CMR) showed acute myocarditis, with mildly impaired LV-function and abundant subepicardial late gadolinium enhancement (LGE). Control CMR after 3 months showed full functional recovery and complete disappearance of LGE. The benefits of SARS-CoV-2 vaccination may significantly exceed the very rare and, in this case, fully reversible adverse effects.

A 21-year-old man presented with fatigue, headache, diffuse myalgia, joint pain, and chest pain, especially during deep inspiration, 2 days after his second severe acute respiratory syndrome coronavirus 2 (SARS-CoV-2) vaccination with BNT162b2 (Pfizer, New York). No history of cardiac disease, risk factors, or family history of cardiovascular or autoimmune diseases was present. Temperature was normal (36.9°C), whereas leukocytosis (11.1/nl) was present with elevated C-reactive protein of 71 mg/L (*normal range* < 5 mg/L). Baseline cardiac troponin was normal (hsTnT of 10 ng/L, *normal range* < 14 ng/L), but ECG showed ST-elevation in precordial leads V2–V5 (Supplementary Figure 1). The coronavirus disease 2019 (COVID-19) swab test by polymerase chain reaction was negative. Baseline echocardiography revealed mildly impaired LV-function (ejection fraction, EF = 45%). Cardiac troponin rose the following days, peaking at 963 ng/L on day 3 of admission.

As a next step, cardiac MR (CMR) imaging was performed using standard balanced steady-state free precession sequences (slice thickness 6 mm for long-axis view, 8 mm for short axis views, matrix 156 × 192, TE = 1.3 ± .5 ms, TR = 45 ± 3 ms, flip angle = 70°) followed by standard mid-ventricular short axis acquisition for T1 mapping [MOLLI 5(3) 3 sequence, slice thickness 8 mm, matrix 218 × 256, TE = 1.33 ms] and T2 mapping (trueFISP sequence, slice thickness 8 mm, matrix 154 × 192, TE = 1.23 ms). After administration of .1 mmol/Kg Dotarem?-gadoterate meglumine, late gadolinium enhancement (LGE) images were acquired using a segmented phase sensitive inversion recovery sequence (PSIR sequence, slice thickness 8 mm, matrix 198 × 224, TE = 1.34 ± 0.3 ms).

The CMR imaging showed acute myocarditis, and mildly impaired LV-function (EF = 49%) [Figures 1A,B, arrowheads depict areas of higher contrast-to-noise ratio in diastolic (A) and systolic (B) SSFP images, performed prior to gadolinium administration, compatible with myocardial oedema]. Abundant subepicardial LGE was detected [arrowheads in Figures 1C,D], whereas native T1 value, measured in the mid-ventricular septum, was within normal range (T1 = 1,000 ms, Figure 1E, arrowheads depict small pericardial effusion) (T1 and T2 images and values are provided in Supplementary Figure 2). Heart failure treatment with bisoprolol 2.5 mg per day was initiated, and the patient exhibited prompt clinical recovery. Control CMR after 3 months showed full functional recovery with an EF of 62% (Figures 1F,G) and disappearance of LGE (Figures 1H,I). T1 value was 990 ms (Figure 1J).

**Figure 1:**
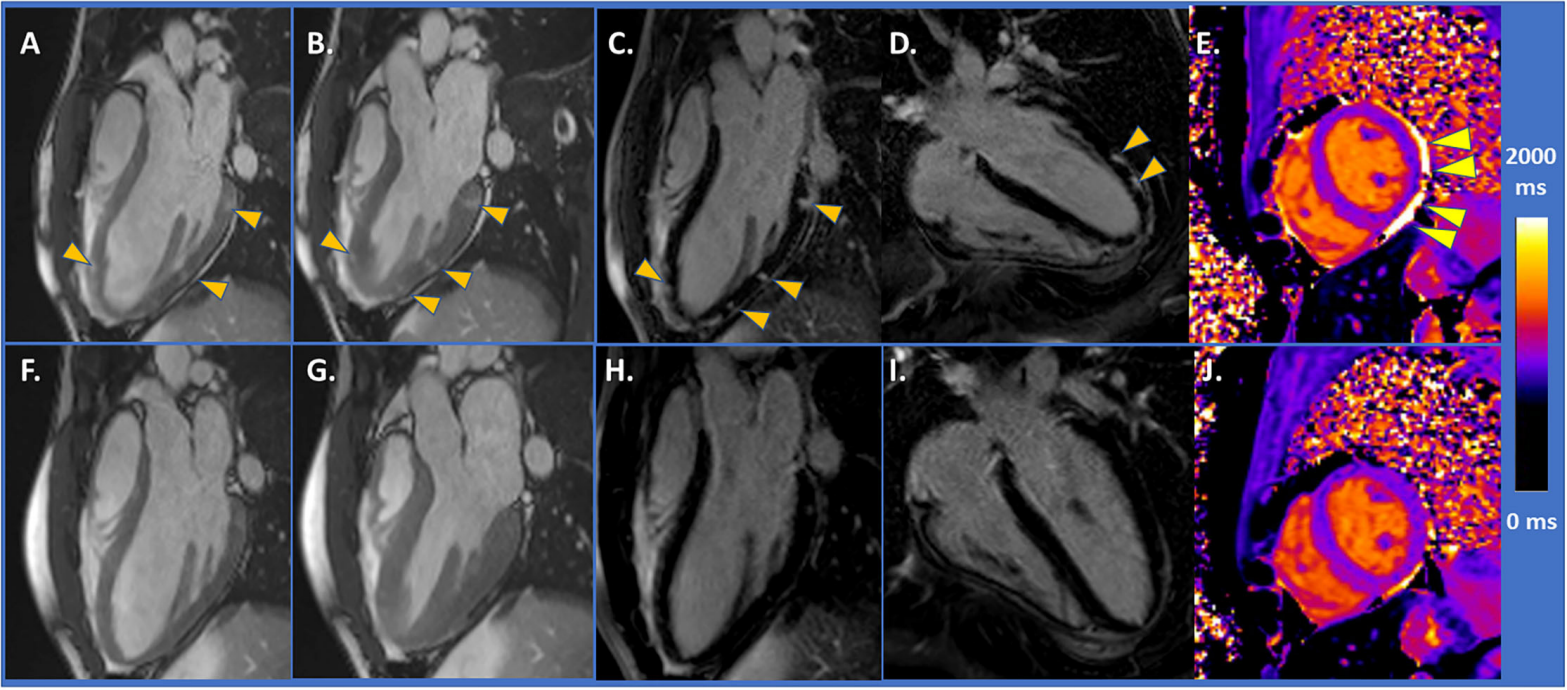
**(A,B)** CMR demonstrated mildly impaired LV-function and areas of higher contrast-to-noise ratio (arrowheads) with SSFP images. **(C,D)** With LGE images, abundant subepicardial LGE was detected (arrowheads). **(E)** Native T1 value was within normal range (T1 = 1,000 ms, arrowheads depicting small pericardial effusion). **(F,G)** Control CMR after 3 months showing full functional recovery and disappearance of LGE in **(H,I)**. **(J)** T1 value of 990 ms.

Recently, a couple of studies demonstrated acute myocarditis in previously healthy young male patients following receipt of mRNA SARS-CoV-2 vaccine (1–3). The acute onset of chest pain 3–5 days after the administration of the second or third dose of the vaccine is a typical feature, reported in the reported cases, suggesting an immune-mediated mechanism. Our case demonstrates complete clinical and functional recovery and disappearance of oedema and LGE in such a young patient with SARS-CoV-2 vaccine related myocarditis. The benefits of SARS-CoV-2 vaccination greatly exceed the very rare and, in this case, fully reversible adverse effects. In this regard, it should be considered that COVID-19 disease can itself contribute to severe myocarditis with biventricular deterioration (4). In addition, CMR imaging emerges as the best non-invasive imaging modality not only for initial diagnosis but also for follow-up of such patients. In this regard, other causes of elevated troponins need to be considered in such patients, including myocardial infarction, takotsubo cardiomyopathy, pulmonary embolism and classical viral, COVID-19 or giant cell myocarditis.

## Data Availability Statement

The raw data supporting the conclusions of this article will be made available by the authors, without undue reservation.

## Ethics Statement

Ethical review and approval was not required for the study on human participants in accordance with the local legislation and institutional requirements. The patients/participants provided their written informed consent to participate in this study.

## Author Contributions

SG and GK performed the procedure and wrote the manuscript. All authors contributed to the article and approved the submitted version.

## Conflict of Interest

The authors declare that the research was conducted in the absence of any commercial or financial relationships that could be construed as a potential conflict of interest.

## Publisher's Note

All claims expressed in this article are solely those of the authors and do not necessarily represent those of their affiliated organizations, or those of the publisher, the editors and the reviewers. Any product that may be evaluated in this article, or claim that may be made by its manufacturer, is not guaranteed or endorsed by the publisher.

## Supplementary Material

Supplementary Figure 1 ECG of the patient demonstrating ST-elevation in the precordial leads V2–V5.

Supplementary Figure 2 T1 and T2 images and the corresponding values at baseline and at 3 months of follow-up.
