# Supplementary material for: Case Report: Disappearance of Late Gadolinium Enhancement and Full Functional Recovery in a Young Patient With SARS-CoV-2 Vaccine-Related Myocarditis
Source: Front Cardiovasc Med. 2022 Mar 8;9:852931. doi: 10.3389/fcvm.2022.852931 (PMC8957274; doi:10.3389/fcvm.2022.852931)
Supplement: Supplementary file 1 [file Presentation_1.PPTX]

## Slide 1
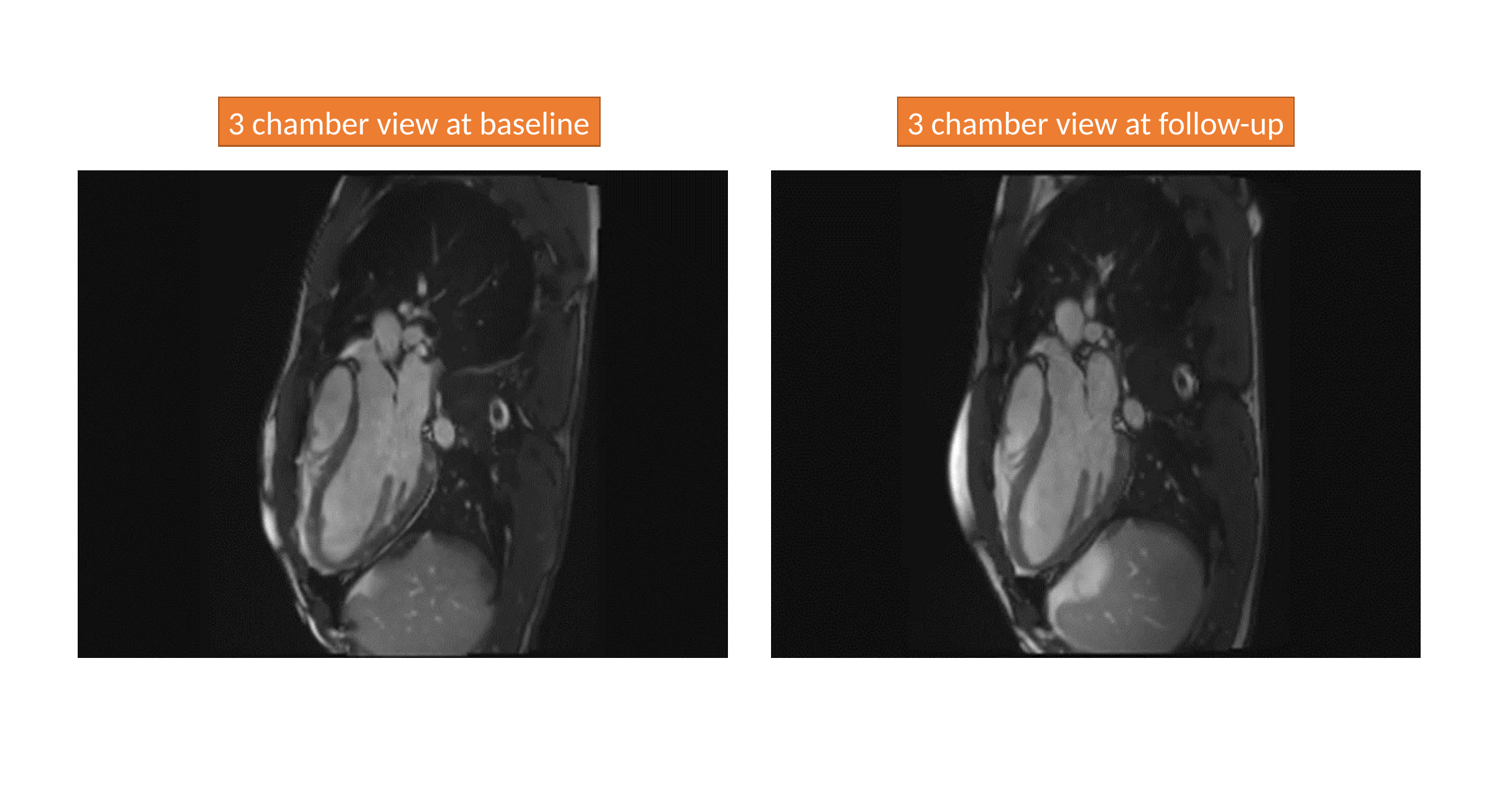

3 chamber view at baseline
3 chamber view at follow-up

## Slide 2
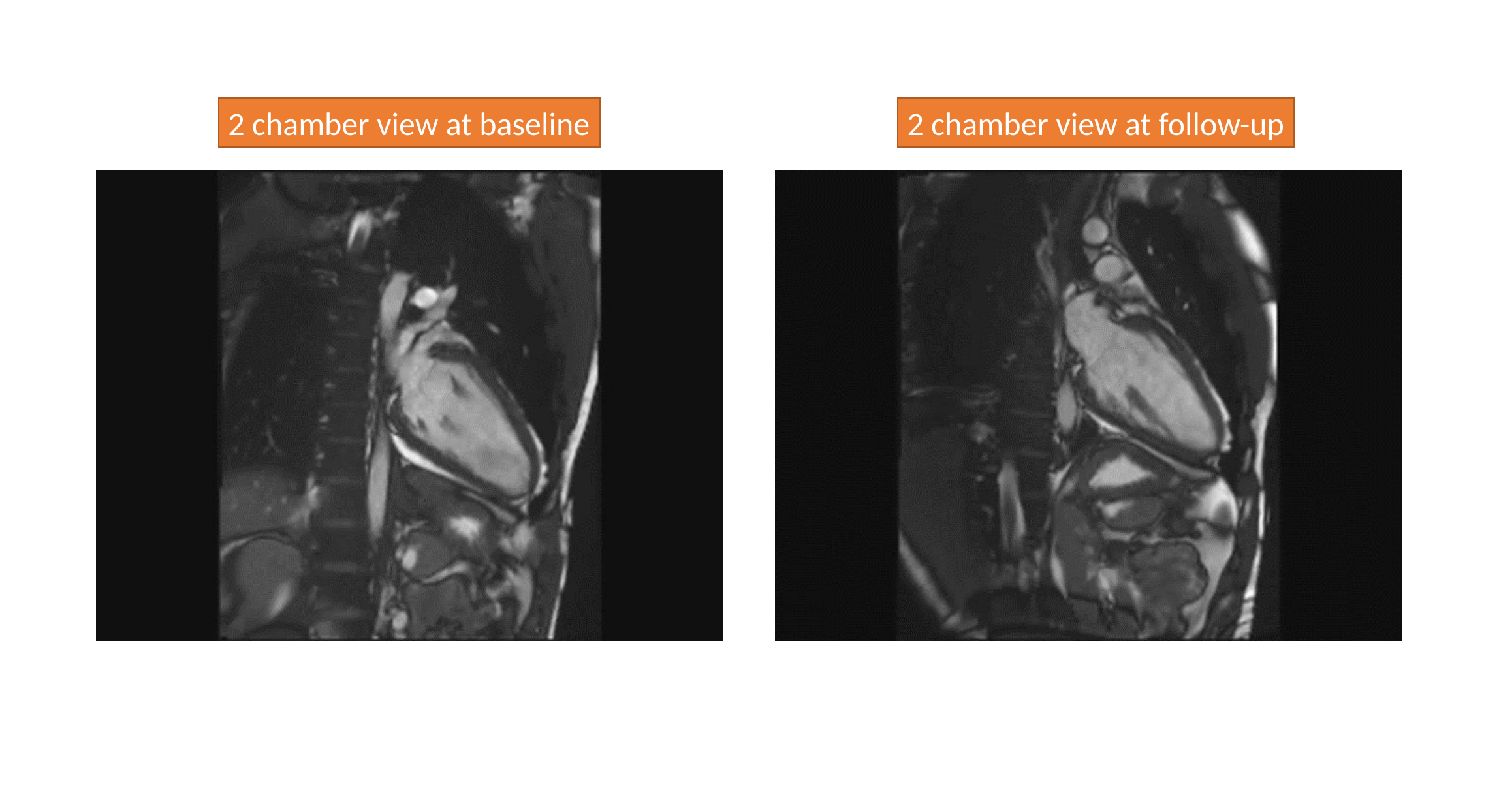

2 chamber view at baseline
2 chamber view at follow-up

## Slide 3
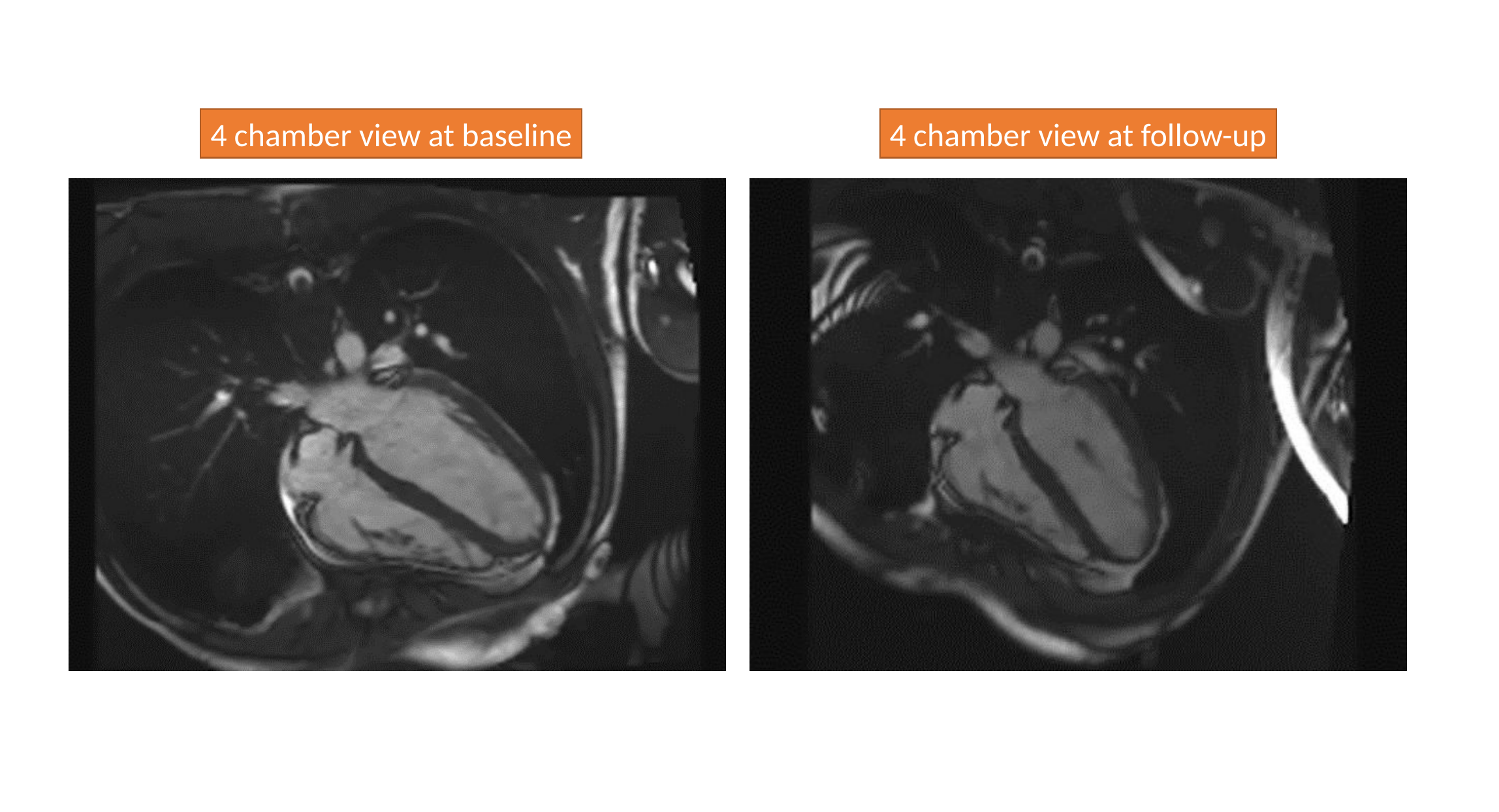

4 chamber view at baseline
4 chamber view at follow-up
