# Supplementary figures and images for: Case Report: Disappearance of Late Gadolinium Enhancement and Full Functional Recovery in a Young Patient With SARS-CoV-2 Vaccine-Related Myocarditis
Source: Front Cardiovasc Med. 2022 Mar 8;9:852931. doi: 10.3389/fcvm.2022.852931 (PMC8957274; doi:10.3389/fcvm.2022.852931)

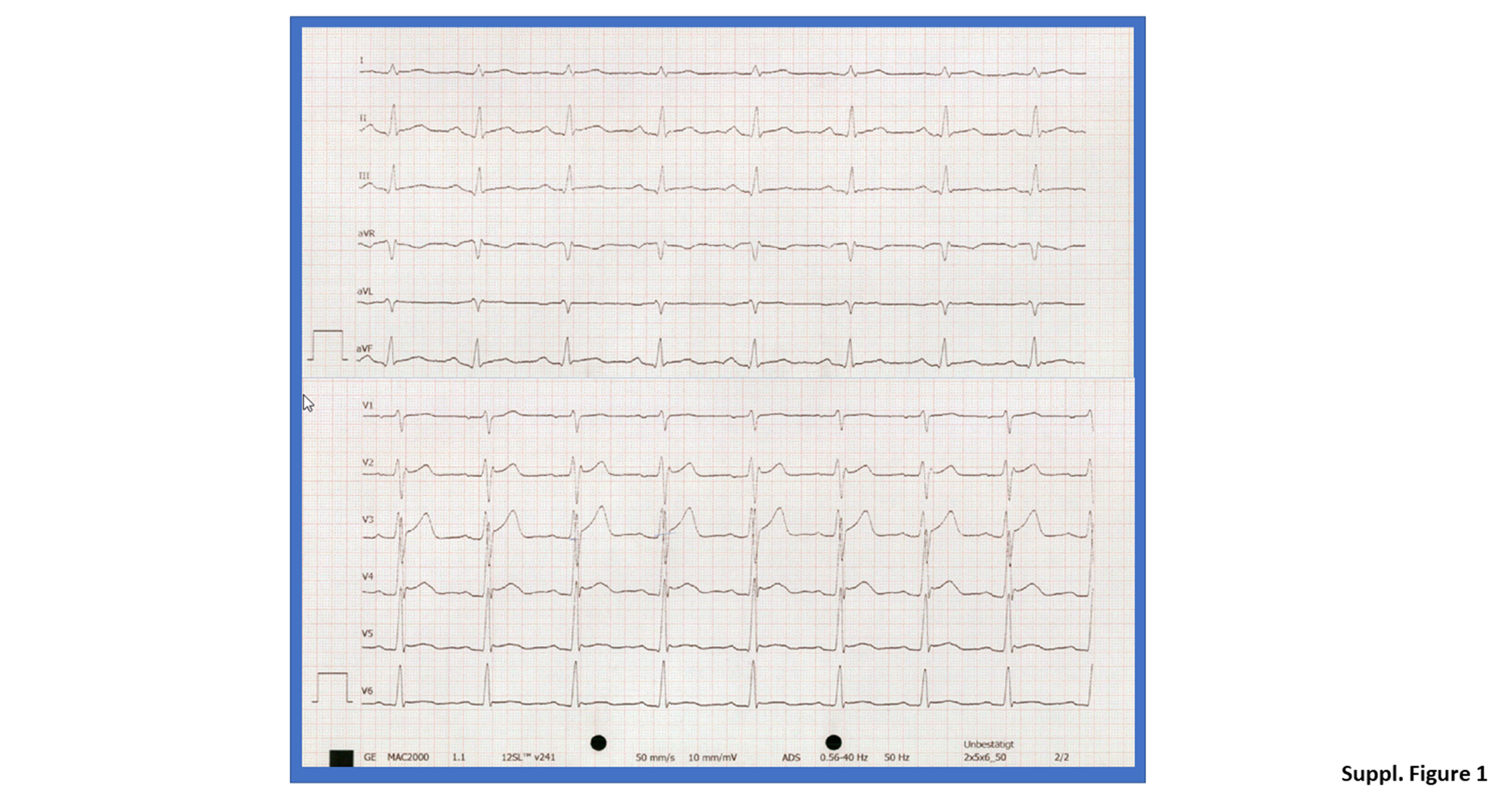

Supplement: Supplementary Figure 1 — ECG of the patient demonstrating ST-elevation in the precordial leads V2–V5. [file Image_1.TIF]

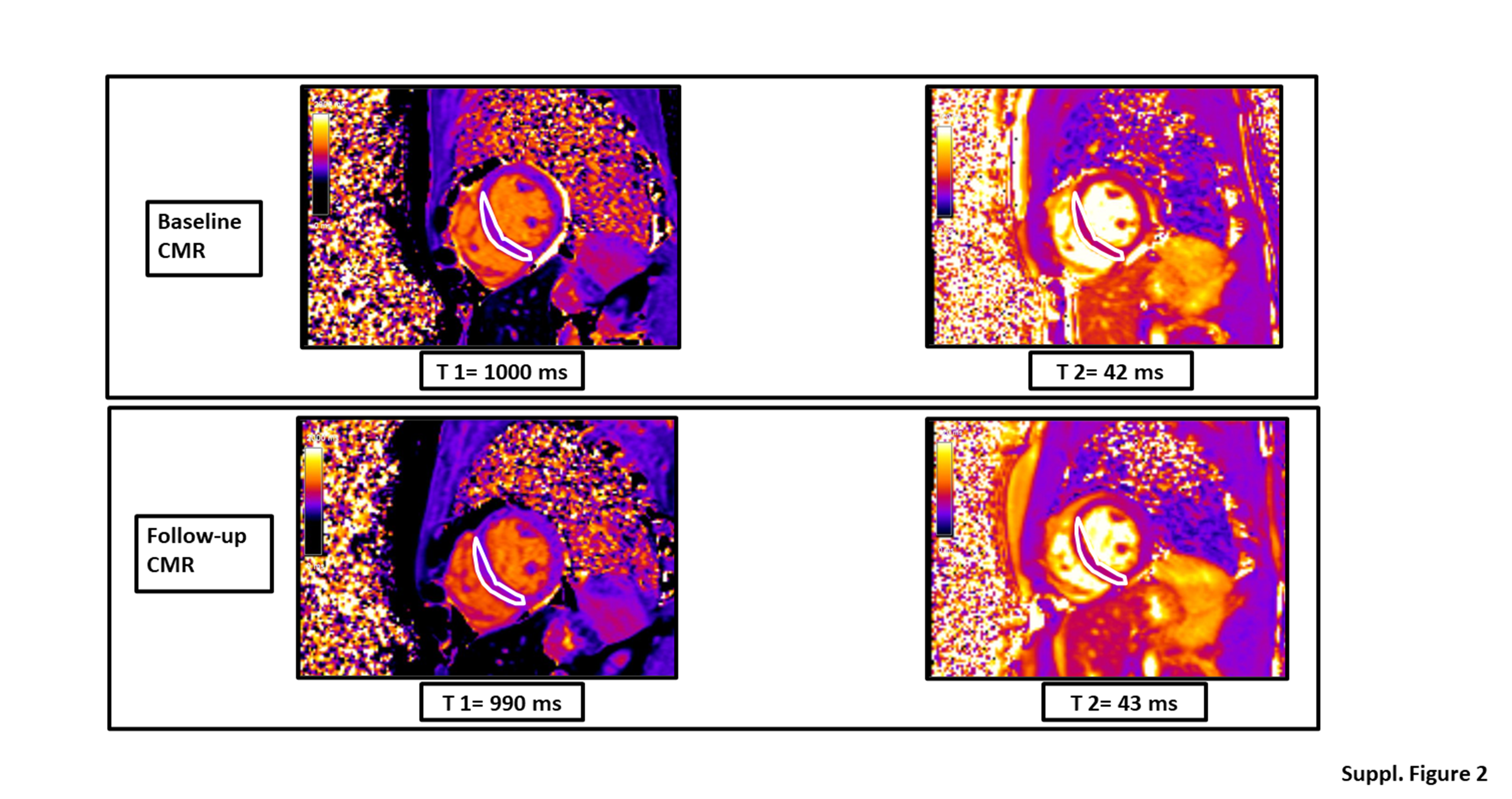

Supplement: Supplementary Figure 2 — T1 and T2 images and the corresponding values at baseline and at 3 months of follow-up. [file Image_2.TIF]
